# Dr. William H. McReynolds

**Published:** 1891-01

**Authors:** 


Dr. William H. McReynolds, of Cincinnati, O., died at his home, November 29, 1890, after a long illness, consecutive to *la grippe* of last Winter. Dr. McReynolds was Surgeon of the 2d Regent Ohio Cavalry during the war, and has been engaged since its close in the active practice of his profession. He was much respected by his professional friends and neighbors in the city of his home, and leaves a widow and three lovely young daughters in bereavement.

